# Hypoxia-induced interstitial transformation of microvascular endothelial cells by mediating HIF-1α/VEGF signaling in systemic sclerosis

**DOI:** 10.1371/journal.pone.0263369

**Published:** 2022-03-01

**Authors:** Jing Mao, Jiexiong Liu, Mei Zhou, Guiqiang Wang, Xia Xiong, Yongqiong Deng

**Affiliations:** 1 Department of Dermatology, The Affiliated Hospital of Southwest Medical University, Luzhou, Sichuan, P.R. China; 2 Department of Infectious Disease, Peking University First Hospital, Beijing, China; University of Texas McGowan Medical School at Houston, UNITED STATES

## Abstract

**Objective:**

The aim of this research was to systematically investigate the effects of endothelial mesenchymal transition (EndMT) induced by hypoxia on the skin microvascular remodeling of systemic sclerosis (SSc) and the underlying mechanism.

**Methods:**

Skin tissues from SSc patients and controls were collected for isobaric tags for the relative and absolute quantification (iTRAQ)-based proteomics and immunohistochemical test. Human microvascular endothelial cell line-1 (HMEC-1) cultured in hypoxic or normal conditions was treated by tamoxifen or bevacizumab.

**Results:**

The iTRAQ-based proteomics indicated a significantly upregulated hypoxia-inducible factor-1 (HIF-1) signal in SSc samples. The immunohistochemical results demonstrated the significant downregulation of CD31, the positive staining of α-smooth muscle actin (α-SMA), HIF-1α, and vascular endothelial growth factor (VEGF-a) in SSc skin tissues, compared with control samples. Consistent with these observations, HMEC-1 cells cultured under hypoxic conditions exhibited a significant decrease in CD31 and VE-cadherin expression, alongside a marked increase in the expression of α-SMA and fibronectin, as well as a distinct upregulation of HIF-1α and VEGF-a, when compared with those under normal conditions. It is noteworthy that the inhibition of HIF-1α by tamoxifen effectively downregulated the hypoxic induction of VEGF-a and α-SMA while rescuing the hypoxic suppression of CD31. In addition, the VEGF-a inhibitor bevacizumab treatment had the same effect on the hypoxic expression of α-SMA and CD31, as a tamoxifen intervention, but did not reduce HIF-1α.

**Conclusion:**

These results suggest that the HIF-1α/VEGF signaling pathway can have a critical role in mediating the effect of hypoxia-induced EndMT on the skin microvascular remodeling of SSc.

## Background

Endothelial-to-mesenchymal cell transition (EndMT) refers to a trans-differentiation process during which endothelial cells (ECs) downregulate the expression of their specific markers, such as CD31 and VE-cadherin, acquiring a phenotype of fibroblast such as α-SMA, fibronectin, and type I collagen [1]. It has been proven that EndMT is a key player in the pathogenesis of tissue fibrosis and fibro-proliferative vasculopathy for various fibrotic diseases [1, 2]. Systemic sclerosis (SSc) is a rare connective tissue disease characterized by autoimmunity, a widespread micro vasculopathy, and fibrosis of the skin and visceral organs [2]. Recently, the role of EndMT in interstitial lung disease associated with SSc has been emphasized [3, 4]. Meanwhile, cells in the intermediate stages of EndMT have been identified in the dermal microvessels of both patients with SSc, and bleomycin-induced and urokinase-type plasminogen activator receptor-deficient mouse models [5]. On the other hand, many studies have confirmed that hypoxia can induce EndMT in vitro and in vivo [6–8]. In the pathogenesis of SSc, reduced capillary blood flow coupled with deficient angiogenesis leads to chronic hypoxia, enforcing a positive feed-forward loop for sustaining vascular remodeling, which further promotes the irreversible extensive tissue fibrosis [2]. This indicates that chronic hypoxia is a prominent trait that contributes to vasculopathy and tissue fibrosis in SSc. However, the process of EndMT induced by hypoxia in the pathogenesis of SSc remains unclear.

Hypoxia-inducible factor-1α (HIF-1α) is a key transcription factor that responds to chronic hypoxia and is directly considered as the "main regulatory factor" of the hypoxia environment [9]. It has been reported that HIF-1α promotes fibrotic disease and its implicated functions include the stimulation of excessive extracellular matrix (ECM), vascular remodeling, and futile angiogenesis, with further exacerbation of chronic hypoxia, the deterioration of patho fibrogenesis, and EndMT [10, 11]. The excessive expression of HIF-1α has been detected in the tissues of systemic sclerosis [12, 13]. In addition, vascular endothelial growth factor (VEGF) is the predominant proangiogenic factor regulated by HIF-1α in hypoxia-related diseases, and the remarkable upregulation of VEGF has been observed in SSc specimens [10, 13]. Recently, the inhibition of the HIF-1α/VEGF signaling pathway has been reported to reverse the anti-angiogenesis effects of chrysophanol [14]. The present study was designed to verify whether hypoxia induces the EndMT in the pathogenesis of SSc that is dependent on HIF-1α/VEGF signaling, which may represent an important and novel therapeutic target for the complications of SSc–associated fibroproliferative vasculopathy and fibrosis.

## Materials and methods

### Patients and tissue samples

Skin tissue samples were obtained from eight patients with diffuse cutaneous SSc and eight age and gender-matched healthy controls by skin biopsy between January 2017 and December 2017 at Affiliated Hospital of Southwest Medical University. The investigators only included patients who met the American College of Rheumatology (ACR)/European League Against Rheumatism (EULAR) 2013 classification criteria for SSc [15]. The control samples were obtained from the normal skin surrounding the nevus when it was removed. All specimens were taken from the fingers, the back of the hand, and the forearm. All the surgical biopsies were performed following the patients’ provision of informed consent and according to Institutional Review Board–approved protocols from the Affiliated Hospital of Southwest Medical University.

### Histopathology and immunohistochemistry

The qualified skin tissue was fixed with 10% formalin overnight and embedded in paraffin. The archived paraffin-embedded samples were sliced into 4-μm sections and histopathologically examined by staining with hematoxylin and eosin. Paraffin-embedded human skin tissue specimens were cut into 5-μm sections and mounted on positively charged slides. The dried sections were deparaffinized in xylene and hydrated through graded alcohols. Antigen retrieval was performed using 0.01 M Sodium Citrate buffer solution pH 6.0 by microwave heating. After immersion in 0.3% hydrogen peroxide to block endogenous peroxidase activity, sections were pre-incubated with 10% normal goat serum to prevent nonspecific binding. The slides were incubated overnight with one of the following primary antibodies: anti-CD31 (ab28364, Abcam, 1:50 dilution, Rabbit polyclonal antibody), anti-α-SMA (ab5694, Abcam, 1:200 dilution, Rabbit polyclonal antibody), anti-HIF-1α (ab51608, Abcam, 1:100 dilution, Rabbit monoclonal antibody), or anti-VEGF-a (ab32152, Abcam, 1:100 dilution, Rabbit monoclonal antibody). The IgG binding was revealed after the incubation with the secondary antibody for one hour. After that, the slides were colored with the diaminobenzidine (DAB) chromogenic agent. Finally, the positive signals of each index were semi-quantitatively counted using ImageJ, and the analysis was carried out.

### Absolute quantitation (iTRAQ)-based proteomics

Overall proteins were extracted from skin tissues of four patients and matched controls and the number of proteins was measured using the bicinchoninic acid (BCA) protein assay kit, following the manufacturer’s instruction. An amount of 1 mg of protein from each sample was deposited in a filtration centricon Microcon YM-30 (Millipore). The washing steps were performed with 8 M of urea, 0.1 M of Tris-HCl, and pH 8.5 buffer. The cysteine residues were blocked with 12 mM of methyl methanethiosulfonate for 30 minutes at room temperature. After that, the proteins were digested by trypsin with an enzyme to a substrate ratio of 1:50 (w/w) at 37°C for 15 hours. Furthermore, the digested peptides were labeled with the iTRAQ Reagent Kit (Applied Biosystems, Foster City, CA, USA). The iTRAQ-labeled samples were analyzed using the nanoACQUITY UPLC system connected to the Q Exactive hybrid quadrupole-Orbitrap mass spectrometer (Thermo Fisher Scientific, USA).

All protein analyses were performed by Majorbio Bio-pharm Technology Co., Ltd. (Shanghai, China). The raw data files were analyzed using the Proteome Discoverer (Thermo Scientific, Version 2.1) against the Homosapien’s database (https://www.uniprot.org/taxonomy/9606). The false discovery rate (FDR) of peptide identification was set as FDR ≤0.01. The functional enrichment analysis was performed for the identified proteins based on the Kyoto Encyclopedia of Genes and Genomes (KEGG) database (http://www.genome.jp/kegg/).

### Cell culture and treatment

The HMEC-1 cell line was obtained from Zhongqiao Xinzhou (Shanghai, China). The cells were cultured in an endothelial cell medium (ECM, Science Cell) containing 5% fetal bovine serum (FBS), 1% penicillin/streptomycin double-antibody, and 1% ECGS endothelial growth factor. The anaerobic gas production bag (Hpebio, Qingdao, China) was used to promote the hypoxia environment, which was placed into the anaerobic culture box. The HMEC-1 cells were treated with hypoxia by culturing in the anaerobic culture box for 72 hours. The phenol blue oxygen indicator was used to monitor the oxygen concentration and hypoxia status. The 5th-8th cell lines of HMEC-1 cells were used for the follow-up experiment.

Tamoxifen, the specific HIF-1α inhibitor purchased from Solarbio (China), was dissolved in dimethyl sulfoxide (DMSO) to construct a solution at a 5-mmol/L concentration. Bevacizumab, the specific VEGF-a inhibitor bevacizumab purchased from MedChemExpress (USA), was dissolved in PBS to construct a solution of 3-mg/ml concentration. The HMEC-1 cells were treated with 5 umol/L of tamoxifen or 0.3 mg/ml of bevacizumab, with DMSO or PBS as the negative control respectively.

### Quantitative reverse transcription-polymerase chain reaction (PCR)

Total RNA was extracted using TRIzol reagent (Invitrogen, Thermo Fisher Scientific, USA), according to the manufacturer’s instructions. β-actin was used as the internal control to detect the mRNA expression of transcription factors HIF-1α, VE-cadherin, fibronectin, CD31, α-SMA, VEGF-a, VEGFR1, and VEGFR2. The EC transcript levels were quantified using the Rever Tra Ace qPCR RT Kit (Code No. FSQ-201; TOYOBO, Japan) on the ABI PRISM 7500 system (Applied Biosystems, USA). The quantification was performed using the 2^-△△CT^ method. The primers used in the PCR analyses were shown in Table 1.

**Table 1:** The primers for PCR analyses.

Genes	Primers	
	Forward	Reverse
HIF-1α	5’-AGCAACTTGAGGAAGTACCATT-3’	5’-AGGTGAACTTTGTCTAGTGCTT-3’
VE-cadherin	5’-AAAGAATCCATTGTGCAAGTCC-3’	5’-CGTGTTATCGTGATTATCCGTG-3’
Fibronectin	5’-AATAGATGCAACGATCAGGACA-3’	5’-GCAGGTTTCCTCGATTATCCTT-3’
CD31	5’-TCGTGGTCAACATAACAGAACT-3’	5’-TTGAGTCTGTGACACAATCGTA-3’
α-SMA	5’-CCGGGAGAAAATGACTCAAATT-3’	5’-CTCAGCAGTAGTAACGAAGGAA-3’
VEGF-a	5’-ATCGAGTACATCTTCAAGCCAT-3’	5’-GTGAGGTTTGATCCGCATAATC-3’
VEGFR1	5’-CAAGATTTGCAGAACTTGTGGA-3’	5’-CTGTCAGTATGGCATTGATTGG-3’
VEGFR2	5’-GGAGCTTAAGAATGCATCCTTG-3’	5’-GATGCTTTCCCCAATACTTGTC-3’

### Western blot

HMEC-1 cells were lysed with RIPA lytic buffer (Beyotime Biotechnology, China). The same number of protein samples were separated by 10% SDS-PAGE and transferred onto an NC membrane (Millipore, USA). After blocking with 5% skim milk, the membranes were incubated overnight at 4°C with one of the following primary antibodies: β-actin (bsm-33036M, bioss, 1:1,000), HIF-1α (ab51608, Abcam, 1:1,000), α-SMA (ab5694, Abcam, 1:500), CD31 (ab28364, Abcam, 1:500), VEGF-a (ab32152, Abcam, 1:500), VE-cadherin (YT4869, Immunoway, 1:500), and fibronectin (YM3137, Immunoway, 1:500). The membranes were washed with PBS and incubated for one hour with the secondary antibody. Finally, the electrochemiluminescence (ECL) reagent was used to visualize them (Millipore, USA). ImageJ was used to capture the protein images.

### Immunofluorescence

HMEC-1 cells were fixed in 4% paraformaldehyde (Biosharp, China) for 30 minutes at room temperature, and permeabilized with 0.2% Triton X-100 (Sigma, USA) and 2% BSA. After blocking with 2% BSA for one hour, these cells were incubated with the primary antibody overnight at 4°C. After that, they were washed in PBS and incubated for one hour with the secondary antibody. Finally, in the dark, the DAPI reagent was added and the images were quickly captured using a fluorescence microscope.

### Statistical analysis

All experiments were repeated at least three times with similar results. The KEGG pathway enrichment analysis was carried out based on the protein-concentrated proteins using the independent development process of Majorbio Bio-pharm Technology Co., Ltd. Fisher’s exact test was employed. With an adjusted P< 0.05, the KEGG pathway was significantly enriched. The KEGG enrichment diagram revealed the relationship between the target protein set, the annotation, and the enrichment of the KEGG pathway. Log2FC was used to show the upregulation and downregulation of proteins. The Z score value was employed to identify the upregulation or downregulation pathways. The other values were expressed as the mean ± SD of at least three independent experiments in triplicate. The statistical differences were assessed by one-way analysis of variance (ANOVA) (Kruskal–Wallis) using the SPSS 17.0 software. A *P*-value of <0.05 was considered statistically significant.

## Results

### Background of participants and iTRAQ-based proteomics

**Fig 1:**
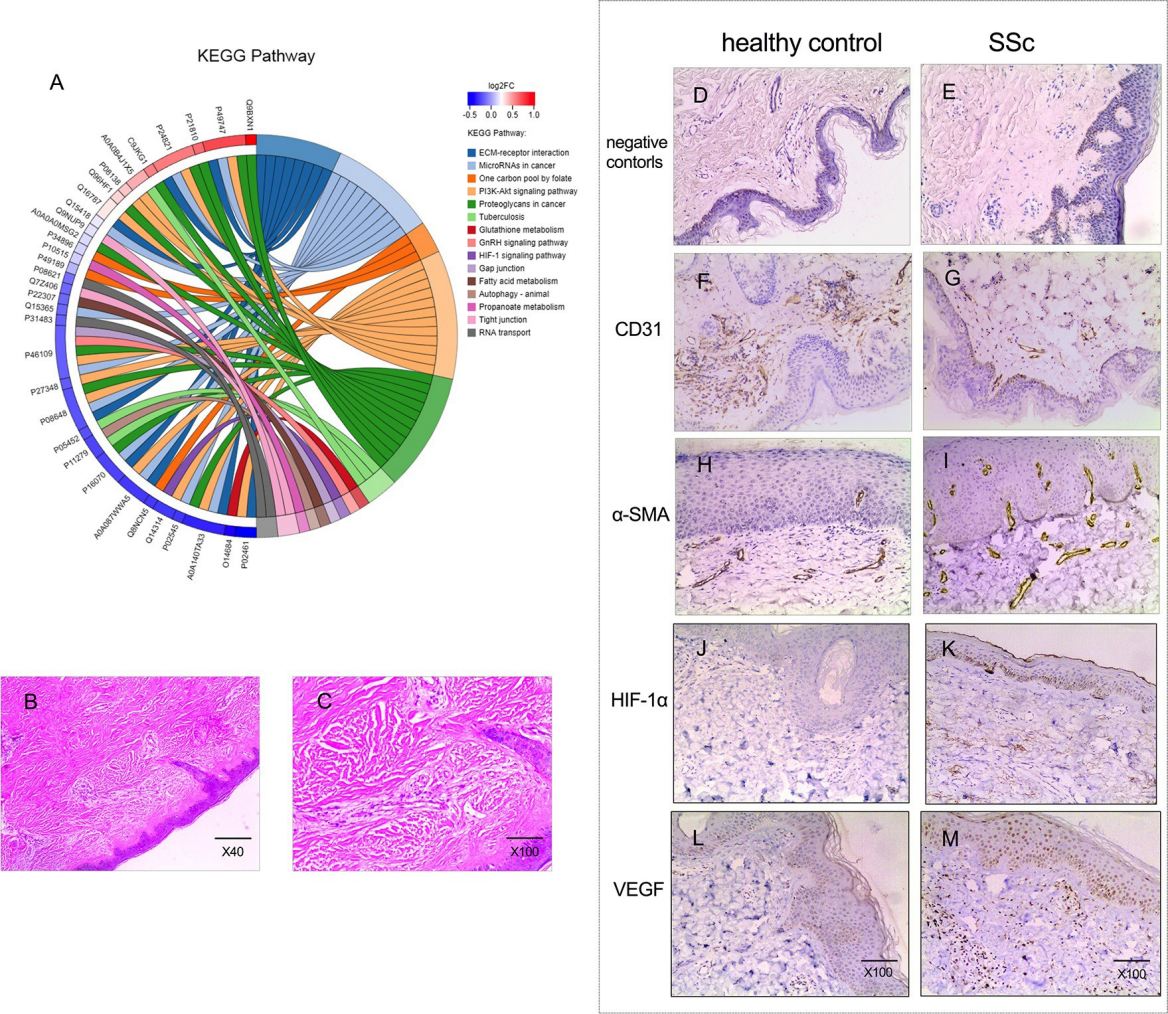
The iTRAQ-based proteomics, and the histopathologic and immunohistologic assessments of skin tissues obtained from patients with SSc. (A) The KEGG pathway enrichment analysis (patients n = 4, controls n = 4). (B and C) Hematoxylin and eosin staining of skin tissues at low and high magnifications from SSc patients. (D and E) Original magnification X100. Negative controls for immunohistochemistry. (F, G, H and I) Immunohistochemical staining of CD31 and α-SMA for both healthy controls (n = 8) and SSc patients (n = 8). The expression of CD31 on patients’ tissue (n = 8) decreased and α-SMA up-regulated, compared with it on healthy controls (n = 8) (J, K, L, and M) Immunohistochemical staining of HIF-1α and VEGF for both healthy controls (n = 8) and SSc patients (n = 8). The HIF-1α and VEGF signaling on the specimen from SSc patients were positive, however, the HIF-1α signaling was not detected and VEGF was lowly expressed on the tissue of healthy controls.

The clinical features of patients with diffuse cutaneous SSc and healthy controls, whose skin tissues were studied, are shown in Table 2. The protein patterns from four patients and four controls were analyzed using the iTRAQ-based quantitative approach. A total of 2,159 proteins were identified. For the amino acid sequences of the identified proteins, the protein dataset was matched to the local database, and 43 proteins were found to be significantly different between SSc patients and controls, with *P* <0.05, FC >1.2, or <0.83, as shown in S1 Table. The functional enrichment analysis was performed for the identified proteins based on the KEGG database. The top 15 up and down pathways are shown in Fig 1A. The left side is the protein, and the right is the name of the KEGG pathway that enriched the target protein. Interestingly, the HIF-1 signal was significantly upregulated in the skin tissues of SSc patients. The Z score for the HIF-1 signaling pathway was 1.0.

**Table 2:** Clinical and demographic characteristics of SSc patients and controls.

Characteristics	SSc, (n = 8)	Controls, (n = 8)	P-value
Age (years)	51±8.49	51±8.49	1.00
Female/Male, n/N	6/2	6/2	1.00
BMI (Kg/m^2^)	21.75±2.63	22.06±1.19	0.77
Duration of disease (years)	1.04±0.82	-	-
Involve organs, n (%)			
Skin	8 (100.0)		
Raynaud phenomenon	7 (87.5)	-	-
Lung	4 (50.0)	-	-
Gastrointestinal tract	3 (37.5)	-	-
Joint	5 (62.5)		
Kidney	1 (12.5)	-	-
Heart	2 (25.0)	-	-
Biopsy site, n (%)			
Finger/Back of hand/ Forearm, n (%)	4 (50.0) / 2 (25.0) / 2 (25.0)	4 (50.0) / 2 (25.0) / 2 (25.0)	1.00
ANA titier, n/N	1:1000 (5/ 8), 1:3200 (2/ 8), 1:320 (1/ 8)	-	-
SCL-70, n/N	+++ (8/ 8)	-	-
Pattern of microangiopathy, n/N	perivascular inflammation (8/ 8), intimal hyperplasia (6/ 8), obliteration of the lumen (2/ 8), vascular loss (7/ 8), microthrombi (1/ 8)	-	-

### The EndMT and activated HIF-1α/VEGF signal were detected in patients with systemic sclerosis

Compared with the controls, all eight skin tissue specimens obtained from the diffuse SSc patients displayed varying degrees of interstitial fibrosis, along with a pattern of infiltration of inflammatory cells around the vessels, marked narrowing, and occlusion of the vessel lumen, and vascular loss, as illustrated in Fig 1B and 1C. Negative controls for immunohistochemistry (Fig 1D and 1E). EC marker CD31 and fibroblast marker α-SMA were used in the immunohistologic analyses to identify the EndMT present in the cutaneous tissue. As expected, CD31 positive cells significantly decreased in SSc tissues, and were detected at sites that lined the vessel lumens when compared with those in control tissues, as illustrated in Fig 1F and 1G. Meanwhile, the positive α-SMA staining of α-SMA was perivascularly shown in the specimens of patients, and on the epidermal cells, but was negative in the control specimen, as illustrated in Fig 1H and 1I.

The HIF-1α/VEGF signal markers were also analyzed by immunohistochemistry. All skin biopsies obtained from SSc patients were both HIF-1α and VEGF positive. However, HIF-1α was not present, and VEGF was lowly expressed in the healthy tissue samples (Fig 1J–1M). The staining of these two markers was observed throughout the keratinocytes of the epidermis, and diffusely distributed throughout the dermis.

### Hypoxia-induced the interstitial transformation of microvascular endothelial cells in vitro

**Fig 2:**
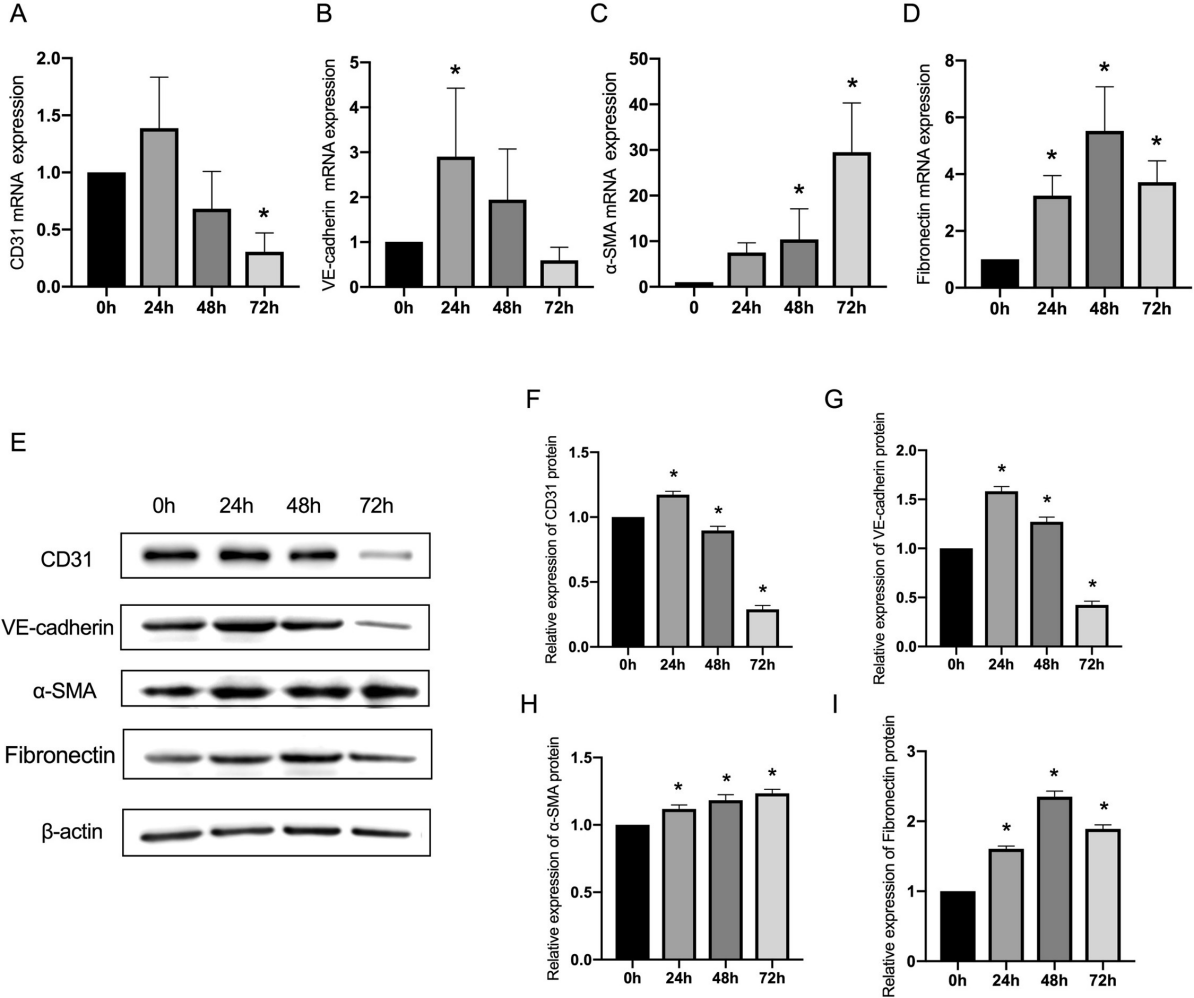
The hypoxia-induced changes of endothelial and fibrotic markers. (A, B, C and D) ECs were exposed to hypoxia for 72 hours, and the mRNA expression was analyzed. (E) The endothelial marker CD31, VE-cadherin, and fibrotic marker α-SMA and fibronectin were detected at protein level by Western blot. (F, G, H and I) Densitometric analyses of the western blot experiments. The above experiments were repeated three times independently. The statistical differences were assessed by one-way analysis of variance (ANOVA) (Kruskal–Wallis), followed by Dunn’s post hoc test. *P<0.05 against the untreated condition. The graph bars show the mean ± SD.

To determine whether hypoxia can induce the conversion of ECs into fibroblast, the EC cell line HMEC-1 was exposed to an anaerobic gas production bag for hypoxia treatment, and the endothelial and fibrotic characteristics were measured. ECs exposed to anaerobic gas exhibited a decrease in mRNA expression for endothelial proteins VE-cadherin and CD31 (Fig 2A and 2B) at every tested hypoxia time point. Furthermore, consistent with the establishment of the fibrotic process, ECs challenged with anaerobic gas exhibited an increase in mRNA levels for fibrotic markers α-SMA and fibronectin (Fig 2C and 2D) at every tested hypoxia time point. CD31 and VE-cadherin protein expression levels were temporarily upregulated after 24 hours of hypoxia, and gradually declined with the extension of hypoxia, as shown in Fig 2E–2G. The protein of α-SMA and fibronectin exhibited a continuous increase with the prolongation of the hypoxia treatment, as shown in Fig 2E, 2H and 2I. The decreasing expression of CD31 and increasing α-SMA with the prolongation of hypoxia treatment were also found by immunofluorescence (see S1 Fig).

The effect of hypoxia on the cellular morphology and polarity of ECs was also investigated. In the absence of hypoxia, HMEC-1 cells presented as circular adherent cells. When these cells were anoxic for 12 hours, there was a small amount of death, but the shape was still full, and the outline was clear. With the duration of hypoxia, the number of cell deaths increased, and HMEC-1 cells lost their typical endothelial cobblestone appearance and acquired a spindle-shaped morphology that was characterized by branches. The changes in cell morphology and shape during hypoxia treatment can be observed in S1 and S2 Figs.

### Hypoxia activated the HIF-1α and VEGF signaling in vitro

**Fig 3:**
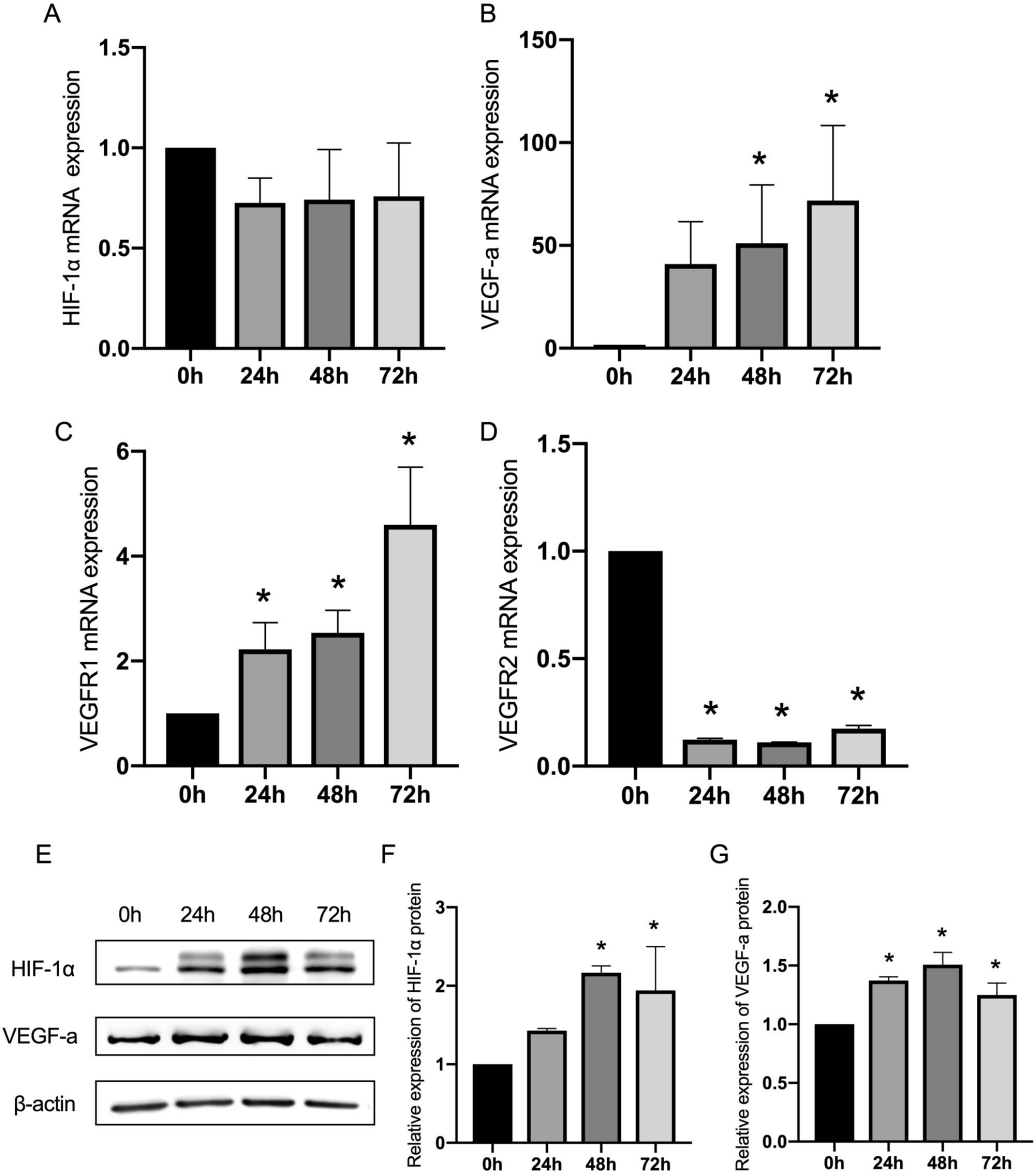
The hypoxia-induced changes of HIF-1α, VEGF-a, VEGFR1 and VEGFR2 at the mRNA and protein level. (A, B, C and D) The ECs were exposed to hypoxia for 72 hours, and the mRNA expression of HIF-1α, VEGF-a, VEGFR1 and VEGFR2 was analyzed. (E) The representative images from the western blot performed for the detection of HIF-1α and VEGF-a. (F and G) Densitometric analyses of the western blot experiments. The above experiments were repeated three times independently. The statistical differences were assessed by one-way analysis of variance (ANOVA) (Kruskal–Wallis), followed by Dunn’s posthoc test. *P<0.05 against the untreated condition. The graph bars show the mean ± SD.

Considering the postulation of the HIF-1α/VEGF signaling pathway, involved in the hypoxia-mediated EndMT, the investigators were prompted to determine whether hypoxia-induced the activation of signal molecules and their receptors at both the mRNA and protein levels. There was no change in HIF-1α mRNA level at the different time points after hypoxia (Fig 3A). However, ECs in the presence of hypoxia exhibited an increase in VEGF-a mRNA level at each of the tested time points (Fig 3B). At the protein level, the detection of HIF-1α and VEGF-a was performed by western blot (Fig 3E–3G) and immunofluorescence (S1 Fig), which indicated the upregulation of both molecules. Meanwhile, the expression of the two receptors of VEGF, Vascular Endothelial Growth Factor Receptor-1 (VEGF-R1) and VEGF-R2 were detected at different time points of hypoxia by real-time quantitative polymerase chain reaction (qRT-PCR). The activation of VEGF-R1 and the inhibition of VEGF-R2 were found, as shown in Fig 3C and 3D.

### The downregulation of HIF-1α inhibits the hypoxia-induced EndMT and VEGF signaling

**Fig 4:**
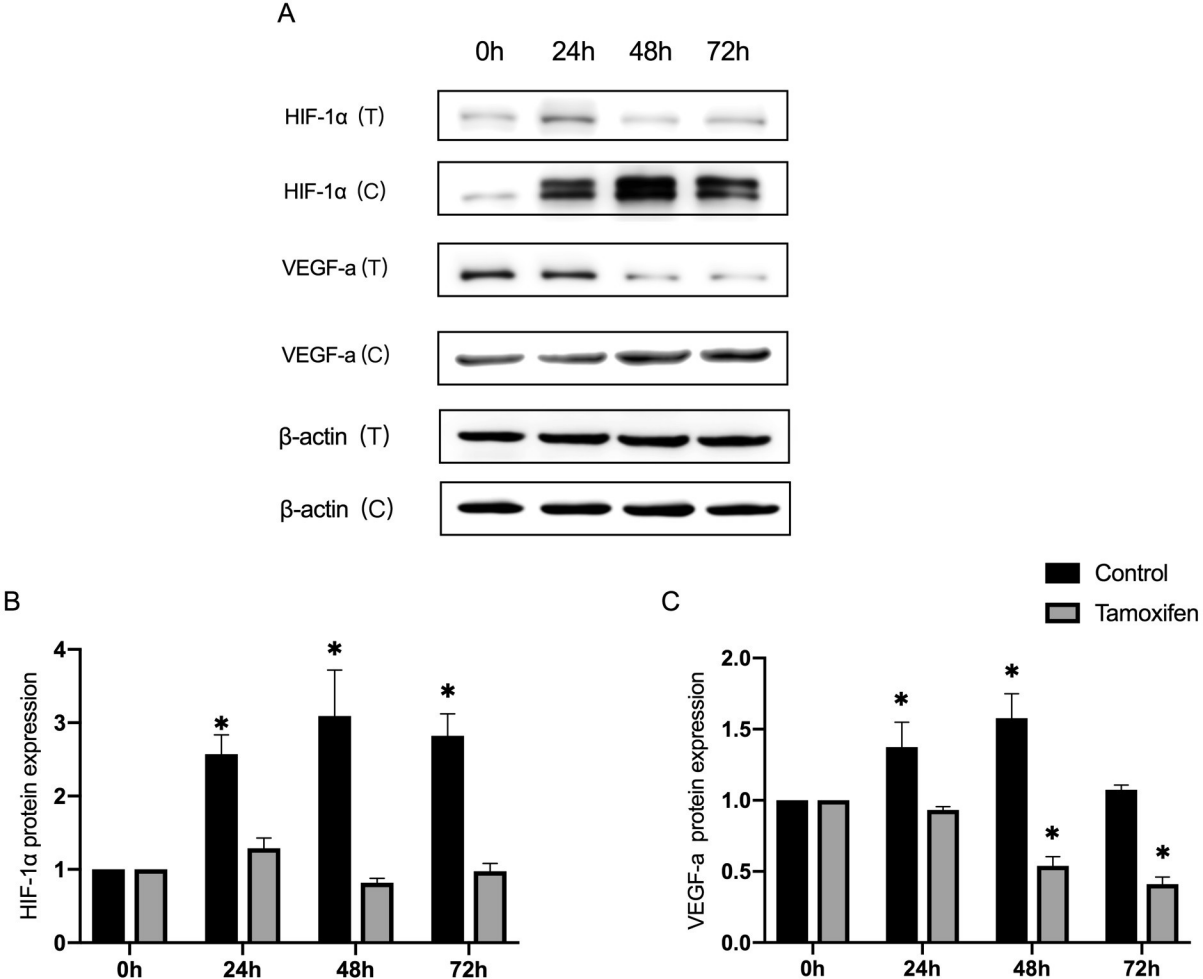
The hypoxia-treated ECs were incubated with tamoxifen, the HIF-1α inhibitor, and the expression of HIF-1α and VEGF-a were detected. (A) The representative images from the western blot performed for the detection of HIF-1α and VEGF-a, with and without tamoxifen treatment. (B and C) Densitometric analyses of the western blot experiments. The above experiments were repeated three times independently. The statistical differences were assessed by one-way analysis of variance (ANOVA) (Kruskal–Wallis), followed by Dunn’s posthoc test. *P<0.05 against the untreated condition. The graph bars show the mean ± SD. The asterisk on top of the bars correlate to the 24, 48 and 72 hours, which represent the comparison with the bars at zero hour.

Although tamoxifen has been reported to be the specific inhibitor of HIF-1α [9], further investigations are needed to determine whether HIF-1α signaling and its probable downstream VEGF signaling induced by hypoxia can be effectively abolished. Hypoxia-treated ECs were incubated with tamoxifen for 72 hours to investigate if signal molecules HIF-1 and VEGF-a might be detected at the protein level. The results revealed that hypoxia-treated ECs with tamoxifen exhibited a sustained decrease in expression of both HIF-1α and VEGF-a (Fig 4A–4C).

**Fig 5:**
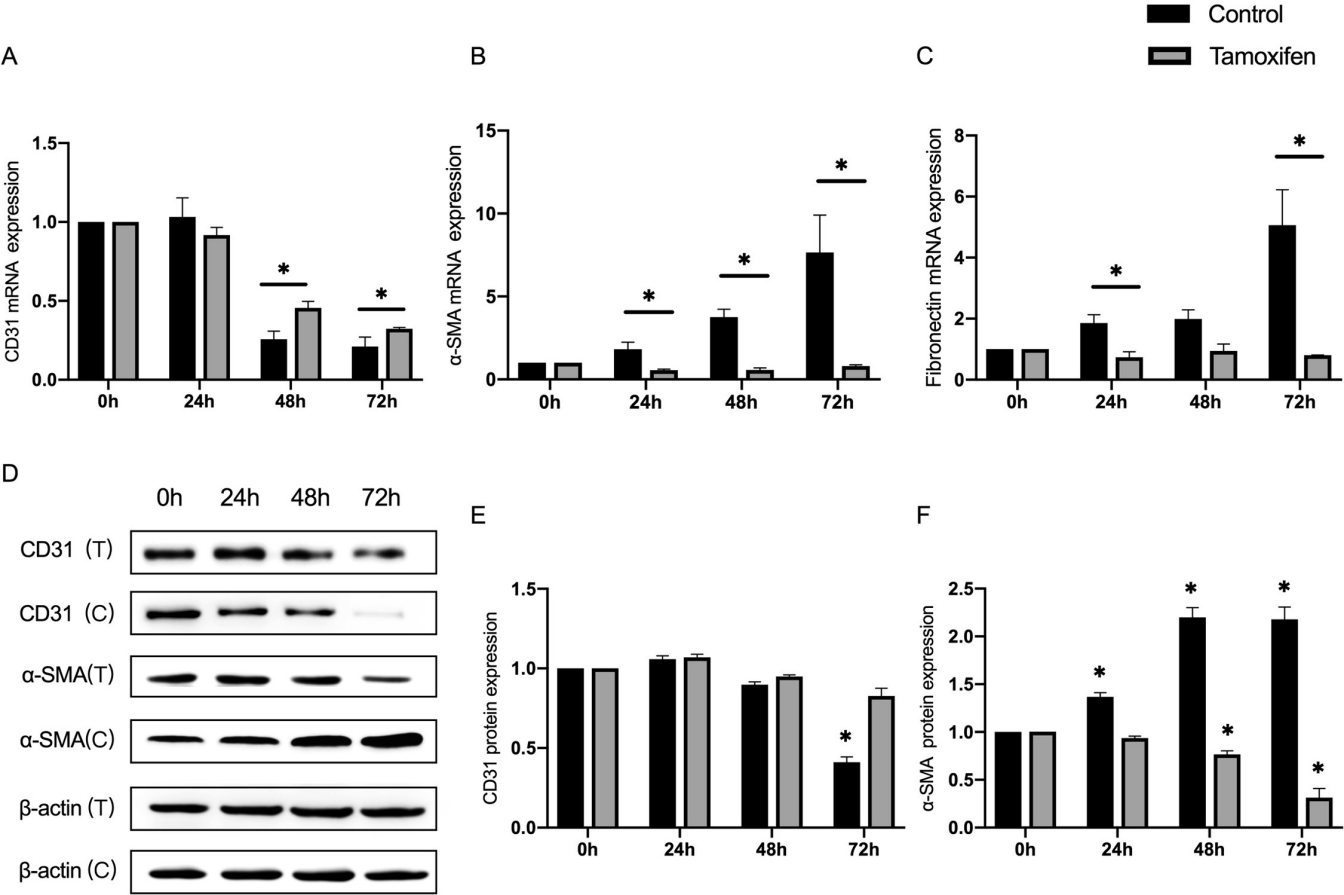
The hypoxia-treated ECs were incubated with tamoxifen, the HIF-1α inhibitor, and the expression of CD31, α-SMA and fibronectin were detected. (A, B and C) The relative expression of CD31, α-SMA and fibronectin at the mRNA level. (D) Representative images from the western blot performed for the detection of CD31 and α-SMA, with and without tamoxifen treatment. (E and F) Densitometric analyses of the western blot experiments. The above experiments were repeated three times independently. The statistical differences were assessed by one-way analysis of variance (ANOVA) (Kruskal–Wallis), followed by Dunn’s posthoc test. *P<0.05 against the untreated condition. The graph bars show the mean ± SD. The asterisk on top of the bars correlate to the 24, 48 and 72 hours, which represent the comparison with the bars of the two groups at the same time point.

Taking into account that hypoxia induces EndMT, and the expression of HIF-1α and VEGF-a, the investigators determined whether the activation of the HIF-1α/VEGF signaling is necessary for the EndMT-induced by hypoxia. They used tamoxifen, the specific inhibitor of HIF-1α/VEGF signaling, and determined the expression of CD31, α-SMA, and fibronectin in hypoxia-treated ECs. The results revealed that the decrease in mRNA expression of EC marker CD31 in hypoxia-treated HMEC-1 cells can be inhibited by tamoxifen intervention (Fig 5A). Furthermore, hypoxia-treated ECs with tamoxifen exhibited a significant decrease in mRNA expression of fibrotic protein α-SMA and fibronectin, which can upregulate in EC induced by hypoxia, without the inhibition of HIF-1α/VEGF signaling (Fig 5B and 5C). The protein levels of CD31 and α-SMA were also detected by western blot. It was found that the expression of EC marker CD31 could not decrease, and even fibroblast marker α-SMA was inhibited in hypoxia-treated ECs while co-culturing with tamoxifen (Fig 5D–5F).

### Hypoxia-induced EndMT is dependent on the activation of HIF-1α/VEGF signaling

**Fig 6:**
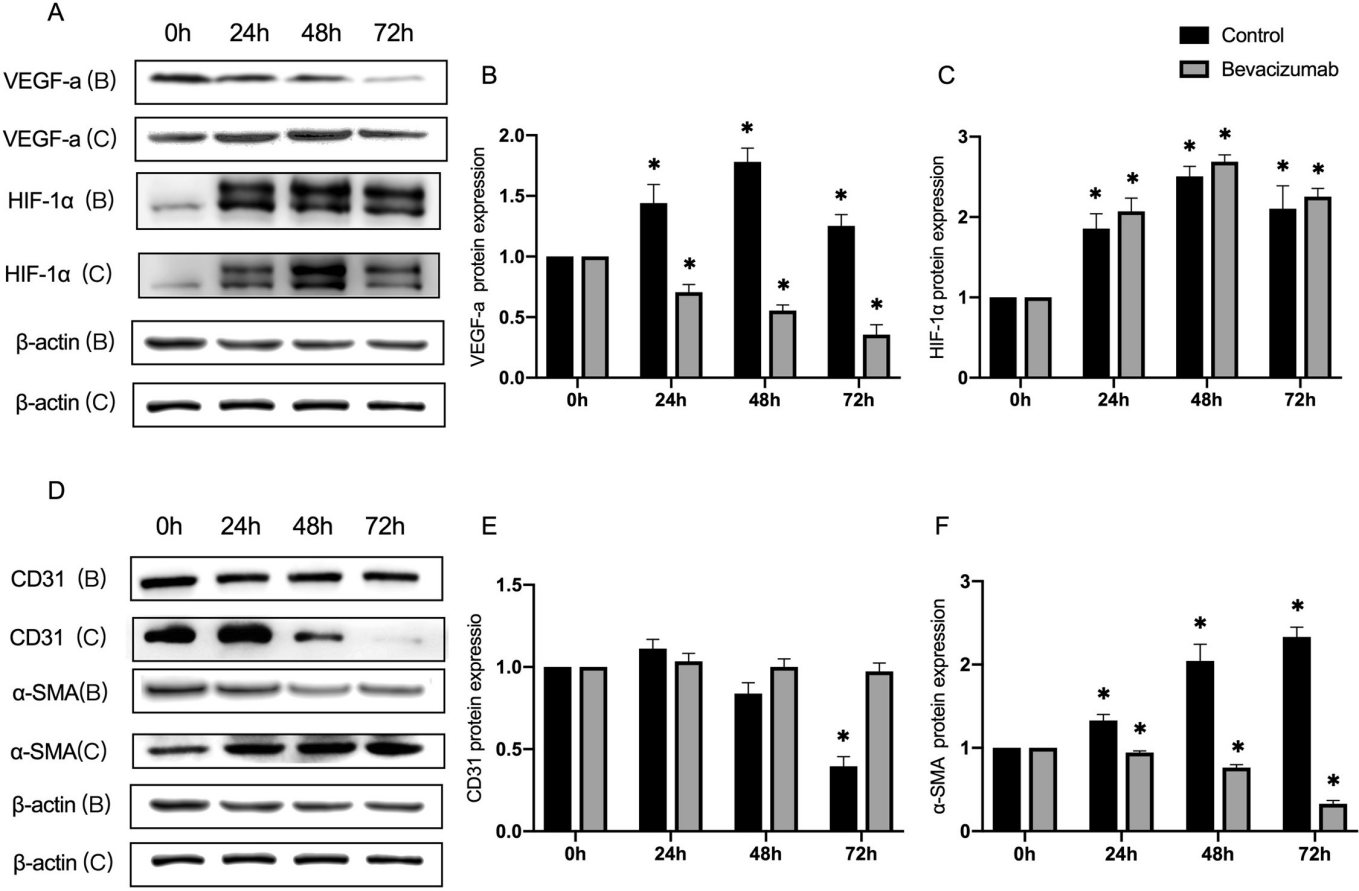
The hypoxia-treated ECs were incubated with bevacizumab, the VEGF-a inhibitor, and the expression of VEGF-a, HIF-1α, CD31 and α-SMA were detected. (A and D) Representative images from the western blot performed for the detection of VEGF-a, HIF-1α, CD31 and α-SMA, with and without bevacizumab treatment. (B, C, E and F) Densitometric analyses of the western blot experiments. The above experiments were repeated three times independently. The statistical differences were assessed by one-way analysis of variance (ANOVA) (Kruskal–Wallis), followed by Dunn’s posthoc test. *P<0.05 against the untreated condition. The graph bars show the mean ± SD. The asterisk on top of the bars correlate to the 24, 48 and 72 hours, which represent the comparison with the bars at zero hour.

Although VEGF-a has been verified as the predominant proangiogenic factor regulated by HIF-1α in hypoxia-related diseases, it remains unknown whether the hypoxia-induced EndMT mediated by the upregulation of HIF-1α signaling was dependent on the activation of VEGF-a. Bevacizumab, the inhibitor of VEGF-a, was used to treat ECs in hypoxia conditions for 72 hours, and HIF-1α, VEGF-a, CD31, and α-SMA were detected at the protein level [16]. VEGF-a can be gradually inhibited by its inhibitor, which can not intervene with the activation of HIF-α induced by hypoxia (Fig 6A–6C). The downregulation of CD31 and upregulation of α-SMA induced by hypoxia can be perfectly rescued by the inhibition of VEGF-a (Fig 6D–6F).

## Discussion

Although the mechanism of SSc vasculopathy is not fully understood, increasing evidence indicates that endothelial injury and subsequent endothelial dysfunction are primary events that trigger the subsequent formation of typical vascular lesions [17]. In clinical practice, Raynaud’s phenomenon (RP), the most common symptom and clinical sign of the disease, is just the result of endothelial injury, which is an important initiating event in SSc [18–20]. In this study, seven out of eight SSc patients clinically manifested as RP, but none of them had digital ulceration, even gangrene, which involved 70% of European SSc cases at the end of the 10-year observation period [21]. The short duration of the disease (1.04±0.82) for patients included in this study partly explains the absence of symptoms of a digital ulcer. Meanwhile, the decreased staining of CD31 and increasing expression of α-SMA around dermis vessels in the specimens of these patients suggested the development of EndMT. It is known that EndMT, a key player in the remodeling of injured vessels, may be reversible, which further accounts for the clinical results, showing that none of the irreversible ischemic tissue injuries were associated with ulceration in this study, and this also possibly offers novel cues for treatment [2].

In addition, it has been proposed that the SSc-related EndMT process has differential pathogenetic roles depending on the type of affected vessels [22, 23]. In arterioles and small arteries, EndMT may lead to an accumulation of profibrotic myofibroblasts in the vessel intima and media, thereby contributing to vessel remodeling, and clinically manifesting as digital ulcers and gangrene of the extremities. When affecting capillary vessels, EndMT may lead to an increase in the number of perivascular myofibroblasts, thereby contributing to tissue fibrosis and a parallel loss of endothelial cells, characterized by micro vessel rarefaction, which clinically promotes the nailfold videocapillaroscopy abnormalities and dermal fibrosis [22]. In this study, most of the patients suffered from clinical RP, high titer of antinuclear antibody (ANA), strongly positive expression of an anti-scl-70 antibody, and pathological skin fibrosis, along with perivascular inflammation, intimal hyperplasia, and vascular loss, while very few cases had pathologically occlusive vasculopathy such as obliteration of the lumen and microthrombi. None of these patients had a digital ulcer. Meanwhile, the decrease in staining of CD31 and increased expression of α-SMA was shown to be mainly around the dermis vessels in the specimens of patients with SSc. The positive HIF-α and VEGF signaling were diffusely distributed in the dermis. These findings support the role of EndMT in skin microangiopathy during active SSc progression, to which HIF-/VEGF signaling may contribute.

HIF-1α is the master transcriptional regulator of the adaptive response to hypoxia, which is the tightly regulated form of HIF-1 and will quickly be hydroxylated and degraded in normoxic conditions by prolyl hydroxylases [24–27]. HIF-1 expression, on the other hand, increases dramatically in hypoxia conditions [28, 29]. HIF-1α has been postulated to be dysregulated in various pathologic conditions, which was also detected throughout the keratinocytes of the epidermis in all skin biopsies obtained from scleroderma patients [13, 30, 31]. In the present study, the positive staining of HIF-1α was not only throughout the keratinocytes of the epidermis but also diffusely distributed in the dermis, which further hints at the important role of hypoxia in the pathogenesis of SSc. In addition, HIF-1α does not affect the change of mRNA. Hypoxia inactivates HIF-1α hydroxylase, which inhibits ubiquitin degradation, and this mainly occurs at the protein level.

VEGF has been reported to be significantly upregulated in all stages of fibrosis and dendritic endothelial cells and it was one of the HIF-1α’s main transcriptional targets in hypoxia-related diseases [13, 32]. Traditionally, VEGF is identified as a key mediator of angiogenesis, which induces the differentiation, proliferation, and migration of endothelial cells, consequently contributing to the formation of vessels through both angiogenesis and vascular remodeling [32]. VEGF exerts its biological functions by binding to its receptors, that is, tyrosine kinase receptors VEGFR1 and VEGFR2. However, unexpectedly, skin tissues of SSc patients with characterized vessel obstruction and loss demonstrate the strong upregulation of VEGF and its two receptors, with more intense staining for VEGFR-2 than for VEGFR-1 [13, 28]. Furthermore, the increase in VEGF and HIF-1α, accompanied by the characteristics of EndMT, were observed in the skin of SSc patients in both previous articles and the present study. Meanwhile, HMEC-1 treated by hypoxic exhibited the downregulated level of CD31, VE-cadherin expression, and there was a marked increase in the expression of α-SMA and fibronectin, along with the increase in expression of HIF-1α and VEGF-a. It is noteworthy that the hypoxia-induced EndMT was effectively reversed by tamoxifen and bevacizumab, the inhibitors of the HIF-1α/VEGF pathway.

The present study has limitations, such as the lack of validation in primary cells. The accumulated results validated at both mRNA and protein levels for cell lines, as well as the findings from scleroderma tissues of patients, confirmed the reliability of these results. Another limitation of the present study was that protein inhibitors are directly used, instead of employing gene-level interventions. However, tamoxifen and bevacizumab have been widely used as effective HIF-α and VEGF inhibitors that are less expensive than genetic intervention.

## Conclusion

The results from the present study provide evidence that hypoxia is a crucial factor in inducing the conversion of ECs into fibroblasts through an HIF-1α/VEGF dependent mechanism that consequently promotes skin microvascular remodeling and fibrosis in SSc. This information would be beneficial for designing novel and improved therapeutic strategies against the complications of SSc associated with fibroproliferative vasculopathy and fibrosis.

## Supporting information

S1 Table The different proteins between SSc patients and controls. (DOC)

S1 Fig Significantly altered expression of the components of CD31, α-SMA, HIF-1α and VEGF-a in endothelial cells under hypoxia examined by immunofluorescence. (PDF)

S2 Fig The influence of hypoxia time on the morphology and polarity of endothelial cells under the microscope. (DOC)

S1 Raw images The original images supporting all blot and gel results. (PDF)

S1 Data The iTRAQ-based proteomics data. (XLS)

## List of abbreviations

EndMT: endothelial mesenchymal transition
SSc: systemic scleroderma
iTRAQ: isobaric tags for relative and absolute quantification
HMEC-1: Human microvascular endothelial cell line-1
HIF-1α: Hypoxia-inducible factor-1α
VEGF: vascular endothelial growth factor
α-SMA: α-smooth muscle actin
EC: endothelial cell
ECM: extracellular matrix
ACR: American College of Rheumatology
EULAR: European League Against Rheumatism
BMI: Body Mass Index
PBS: phosphate buffer saline
DAB: Diaminobenzidine
BCA: bicinchoninic acid
KEGG: Kyoto Encyclopedia of Genes and Genomes
ECM: Endothelial Cell Medium
FBS: fetal bovine serum
ECGS: endothelial growth factor
DMSO: dimethyl sulfoxide
ECL: electrochemiluminescence
ANOVA: Analysis of Variance
VEGFR: Vascular Endothelial Growth Factor Receptor
qRT-PCR: Real-time Quantitative polymerase chain reaction
RP: Raynaud’s phenomenon
ANA: antinuclear antibody
ET-1: Endothelin-1
