# Supplementary material for: Hypoxia-induced interstitial transformation of microvascular endothelial cells by mediating HIF-1α/VEGF signaling in systemic sclerosis
Source: PLoS One. 2022 Mar 1;17(3):e0263369. doi: 10.1371/journal.pone.0263369 (PMC8887755; doi:10.1371/journal.pone.0263369)
Supplement: S1 Table — (DOC) [file pone.0263369.s001.doc]

**S1 Table.** The different proteins between SSc patients and controls with P<0.05 and FC>1.2 or FC<0.83

| Accession | Protein name | FC value | P  value | Relative expression mean | | Subcellular Location | Number of peptides  matched | Molecular  weight  (kDa) |
| --- | --- | --- | --- | --- | --- | --- | --- | --- |
|  |  |  |  | ssc | control |  |  |  |
| P23141 | Liver carboxylesterase 1 | 0.63 | 0.034 | 1.229 | 1.94 | mitochondrial | 1 | 62.481 |
| P28288 | ATP-binding cassette sub-family D member 3 | 0.81 | 0.027 | 1.089 | 1.34 | mitochondrial | 1 | 75.428 |
| Q9Y6C9 | Mitochondrial carrier homolog 2 | 0.77 | 0.005 | 0.987 | 1.282 | plasma membrane | 1 | 33.309 |
| K7ERG9 | Complement factor D | 0.69 | 0.00 | 0.9715 | 1.396 | cytoplasmic | 1 | 27.845 |
| P98179 | RNA-binding protein 3 | 0.74 | 0.00 | 1.007 | 1.348 | nuclear | 1 | 17.16 |
| D3YTG3 | Target of Nesh-SH3 | 0.69 | 0.032 | 0.9672 | 1.39 | ER | 3 | 195.204 |
| Q8NCN5 | Pyruvate dehydrogenase phosphatase regulatory subunit, mitochondrial | 0.82 | 0.007 | 1.021 | 1.24 | mitochondrial | 1 | 99.301 |
| Q8NGA1 | Olfactory receptor 1M1 | 0.82 | 0.006 | 1.089 | 1.323 | plasma membrane | 1 | 34.816 |
| P50238 | Cysteine-rich protein 1 | 0.79 | 0.035 | 1.058 | 1.335 | cytoplasmic | 3 | 8.527 |
| P58107 | Epiplakin OS=Homo sapiens | 0.77 | 0.004 | 1.041 | 1.344 | cytoplasmic | 60 | 555.316 |
| P26447 | Protein S100-A4 | 0.73 | 0.000 | 0.9875 | 1.355 | cytoplasmic | 5 | 11.721 |
| P02461 | Collagen alpha-1(III) chain | 0.68 | 0.045 | 0.9295 | 1.363 | cytoplasmic | 11 | 138.479 |
| P60903 | Protein S100-A10 | 0.75 | 0.035 | 0.9975 | 1.322 | cytoplasmic | 4 | 11.196 |
| Q9NRN5 | Olfactomedin-like protein 3 | 0.82 | 0.025 | 1.036 | 1.265 | cytoplasmic | 7 | 45.981 |
| P14209 | CD99 antigen | 0.69 | 0.006 | 1.038 | 1.505 | extracellular | 1 | 18.836 |
| Q9H299 | SH3 domain-binding glutamic acid-rich-like protein 3 | 0.72 | 0.013 | 0.944 | 1.31 | cytoplasmic | 5 | 10.431 |
| P04083 | Annexin A1 OS=Homo sapiens | 0.82 | 0.045 | 1.001 | 1.216 | cytoplasmic | 16 | 38.69 |
| C9JRZ6 | MICOS complex subunit | 0.73 | 0.016 | 0.9478 | 1.303 | cytoplasmic | 2 | 26.737 |
| O14684 | Prostaglandin E synthase | 0.75 | 0.024 | 0.9645 | 1.287 | Golgi apparatus | 1 | 17.091 |
| P19827 | Inter-alpha-trypsin inhibitor heavy chain H1 | 0.81 | 0.026 | 0.9215 | 1.142 | ER | 8 | 101.326 |
| Q14314 | Fibroleukin | 1.22 | 0.019 | 0.887 | 1.078 | plasma membrane | 1 | 50.197 |
| Q53TN4 | Cytochrome b reductase 1 | 0.76 | 0.049 | 0.888 | 1.17 | plasma membrane | 2 | 31.621 |
| A0A140TA33 | Tenascin-X | 0.79 | 0.032 | 0.8932 | 1.13 | plasma membrane | 45 | 457.989 |
| P02545 | Prelamin-A/C | 0.82 | 0.012 | 0.9 | 1.097 | cytoplasmic | 44 | 74.095 |
| A0A087WWA5 | Tenascin-X | 0.83 | 0.028 | 0.837 | 1.014 | extracellular | 3 | 165.435 |
| G3V4U0 | Fibulin-5 | 1.65 | 0.035 | 1.272 | 0.772 | extracellular | 4 | 50.847 |
| Q96HF1 | Secreted frizzled-related protein 2 | 1.27 | 0.017 | 1.1 | 0.868 | extracellular | 2 | 33.468 |
| C9JKG1 | Biglycan (Fragment) | 1.45 | 0.029 | 1.077 | 0.7283 | extracellular | 1 | 26.728 |
| P08138 | Tumor necrosis factor receptor superfamily member 16 | 1.30 | 0.004 | 0.9265 | 0.7123 | plasma membrane | 1 | 45.154 |
| P28062 | Proteasome subunit beta type-8 | 1.32 | 0.011 | 0.8422 | 0.6385 | mitochondrial | 1 | 30.335 |
| O43823 | A-kinase anchor protein 8 | 1.33 | 0.041 | 0.825 | 0.6182 | nuclear | 1 | 76.061 |
| A0A0A0MRM9 | Nucleolar and coiled-body phosphoprotein 1 | 1.28 | 0.041 | 0.8562 | 0.669 | nuclear | 2 | 74.61 |
| P04264 | Keratin, type II cytoskeletal 1 | 1.27 | 0.044 | 0.9068 | 0.718 | cytoplasmic | 39 | 65.999 |
| Q9BXN1 | Asporin | 2.04 | 0.019 | 1.09 | 0.534 | ER | 13 | 43.389 |
| F8VW96 | Cysteine and glycine-rich protein 2 | 1.27 | 0.011 | 0.9172 | 0.7222 | cytoplasmic | 1 | 26.719 |
| Q16787 | Laminin subunit alpha-3 | 1.23 | 0.007 | 1.01 | 0.823 | plasma membrane | 4 | 366.414 |
| P24821 | Tenascin | 1.49 | 0.006 | 1.002 | 0.6725 | plasma membrane | 25 | 240.7 |
| Q6E0U4 | Dermokine | 1.40 | 0.008 | 0.8988 | 0.6415 | extracellular | 4 | 47.054 |
| Q9HA64 | Ketosamine-3-kinase | 1.48 | 0.005 | 1.029 | 0.6935 | cytoplasmic | 1 | 34.39 |
| P49747 | Cartilage oligomeric matrix protein | 1.65 | 0.002 | 1.08 | 0.6547 | extracellular | 8 | 82.808 |
| P33993 | DNA replication licensing factor MCM7 | 1.28 | 0.014 | 0.8502 | 0.6662 | cytoplasmic | 1 | 81.257 |
| P21810 | Biglycan | 1.54 | 0.028 | 1.015 | 0.659 | cytoplasmic | 7 | 41.628 |
| A0A0B4J1X5 | Immunoglobulin heavy variable 3-74 | 1.36 | 0.033 | 0.8368 | 0.6172 | plasma membrane | 1 | 12.831 |
