# Supplementary figures and images for: Hypoxia-induced interstitial transformation of microvascular endothelial cells by mediating HIF-1α/VEGF signaling in systemic sclerosis
Source: PLoS One. 2022 Mar 1;17(3):e0263369. doi: 10.1371/journal.pone.0263369 (PMC8887755; doi:10.1371/journal.pone.0263369)

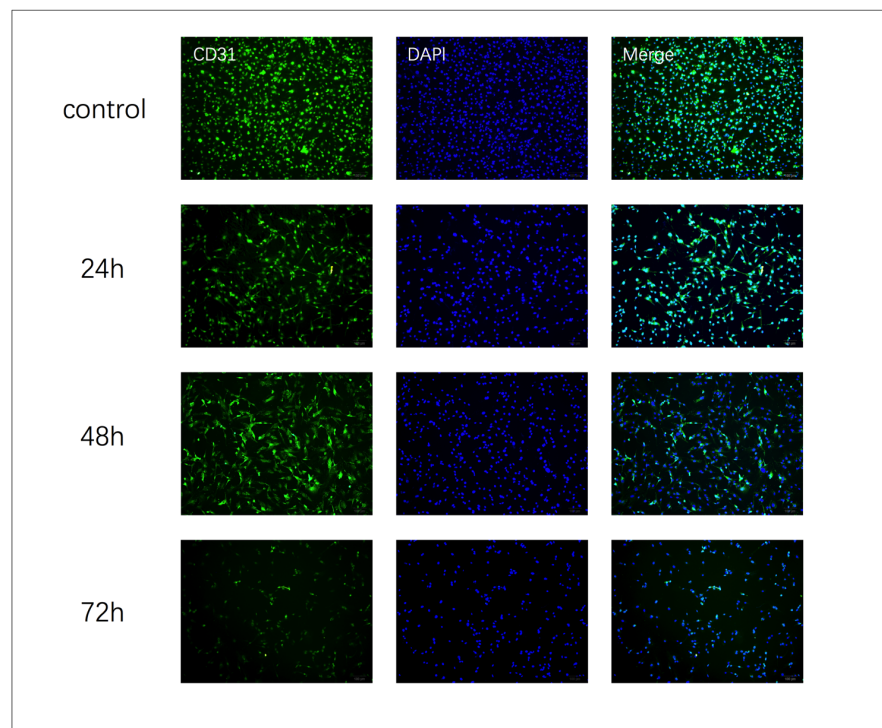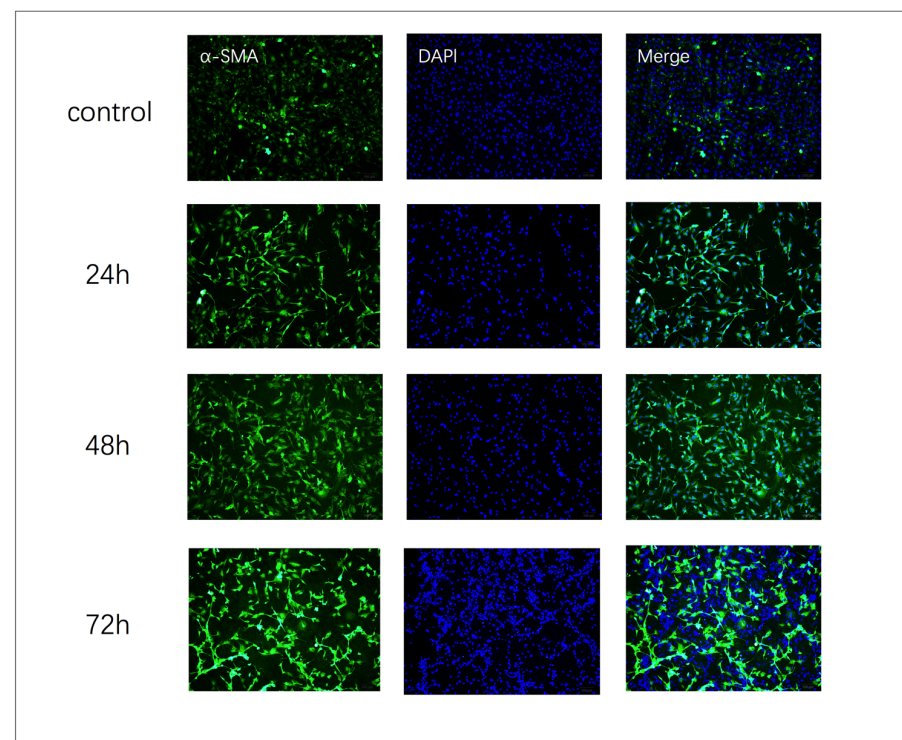

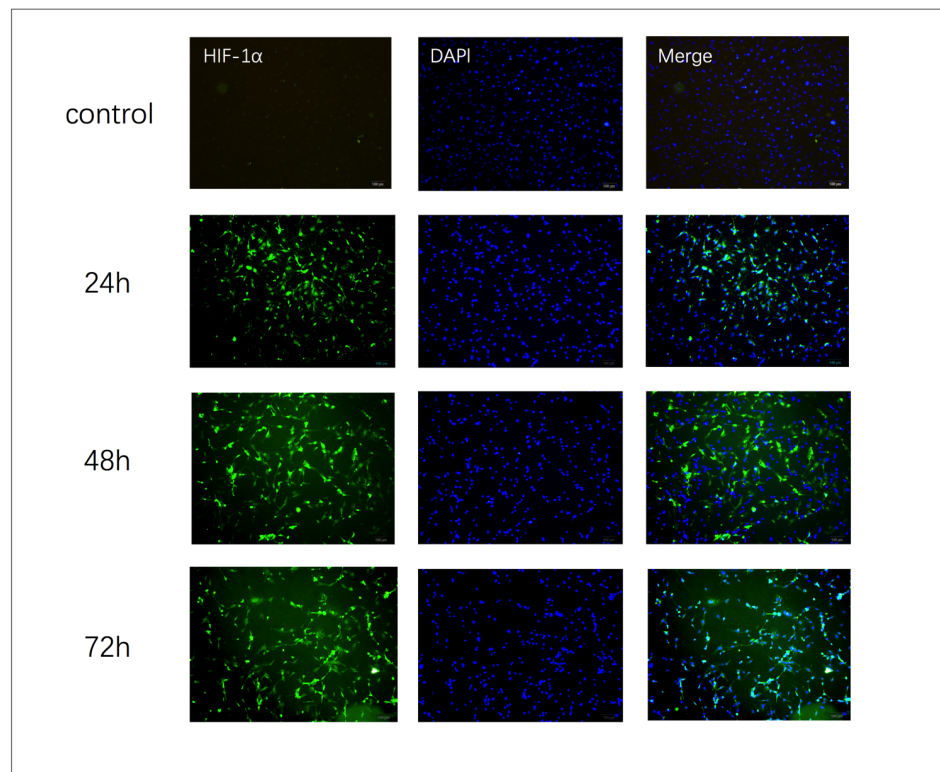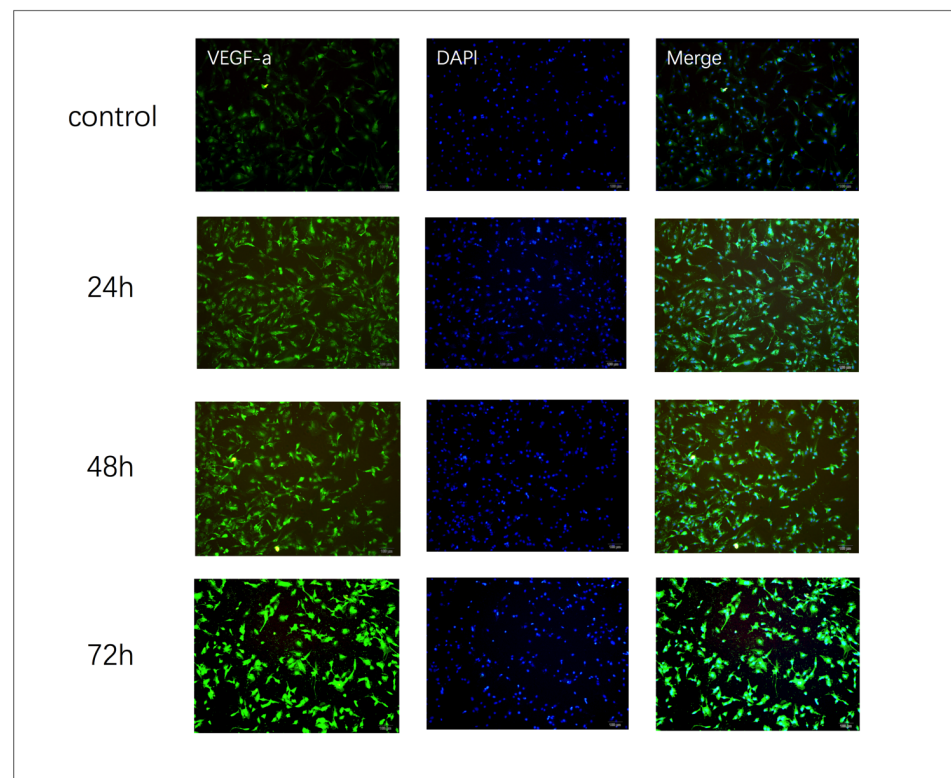

Supplement: S1 Fig — (PDF) [file pone.0263369.s002.pdf]

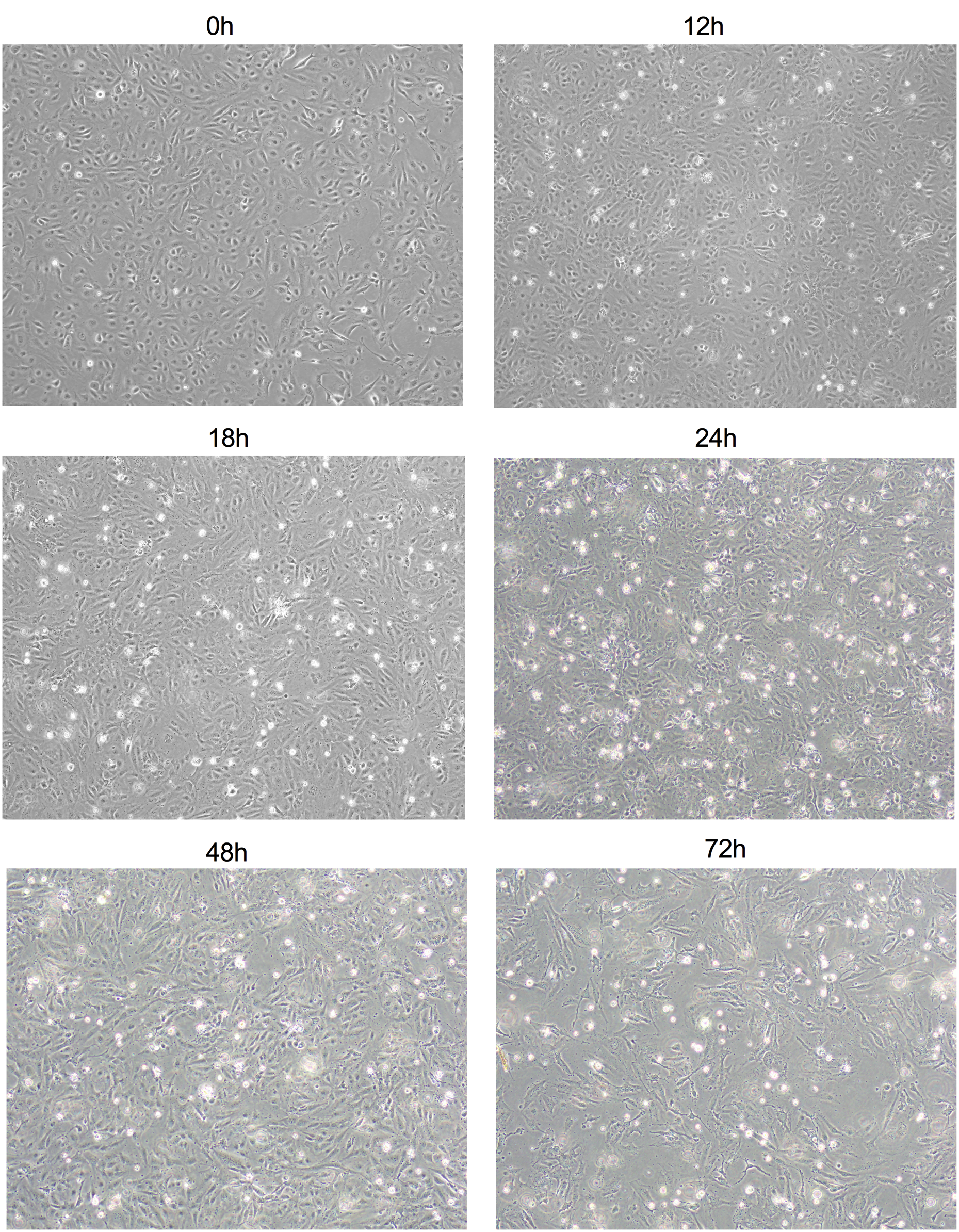

Supplement: S2 Fig — (DOC) [file pone.0263369.s003.doc]

Fig 2

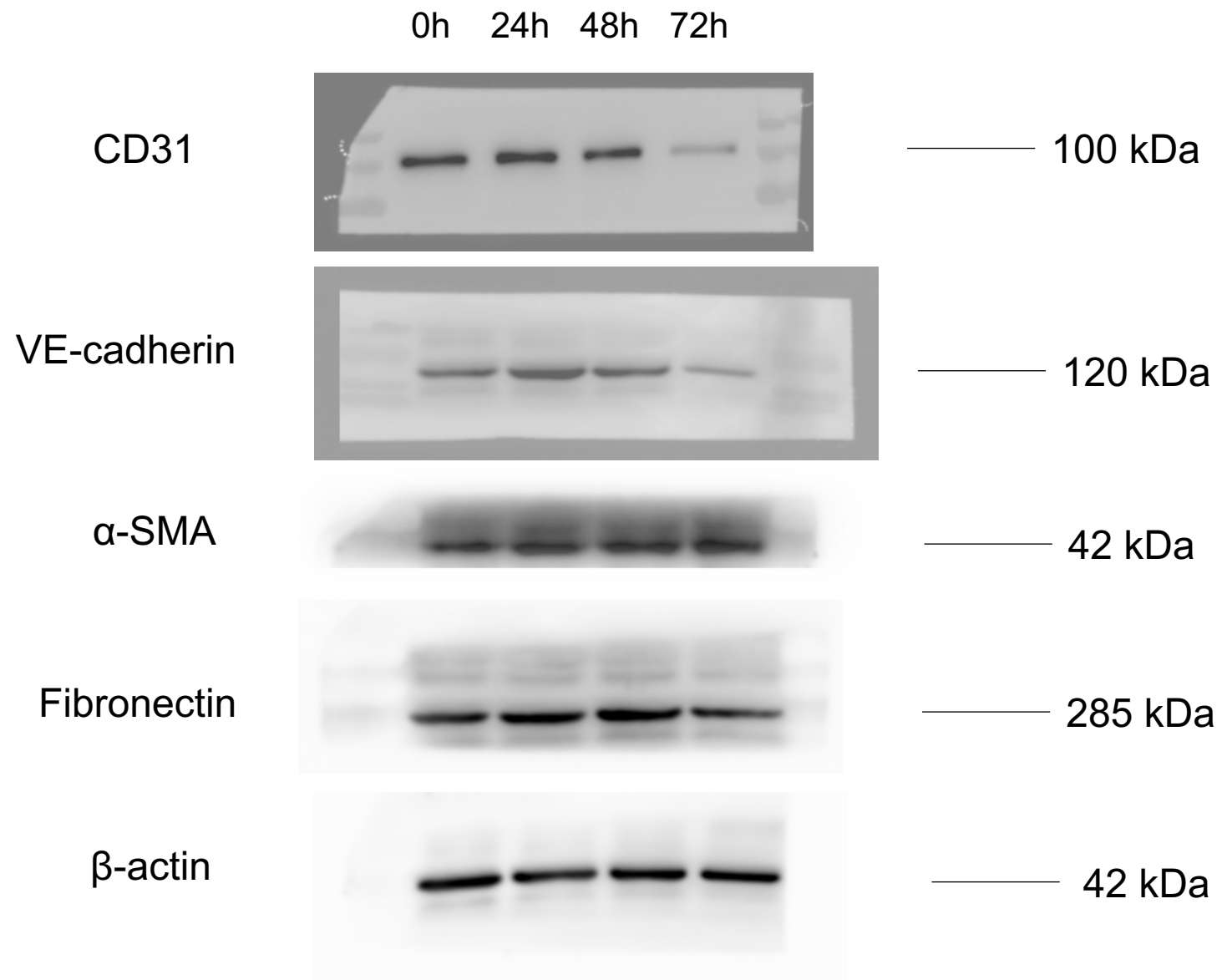

Fig 3

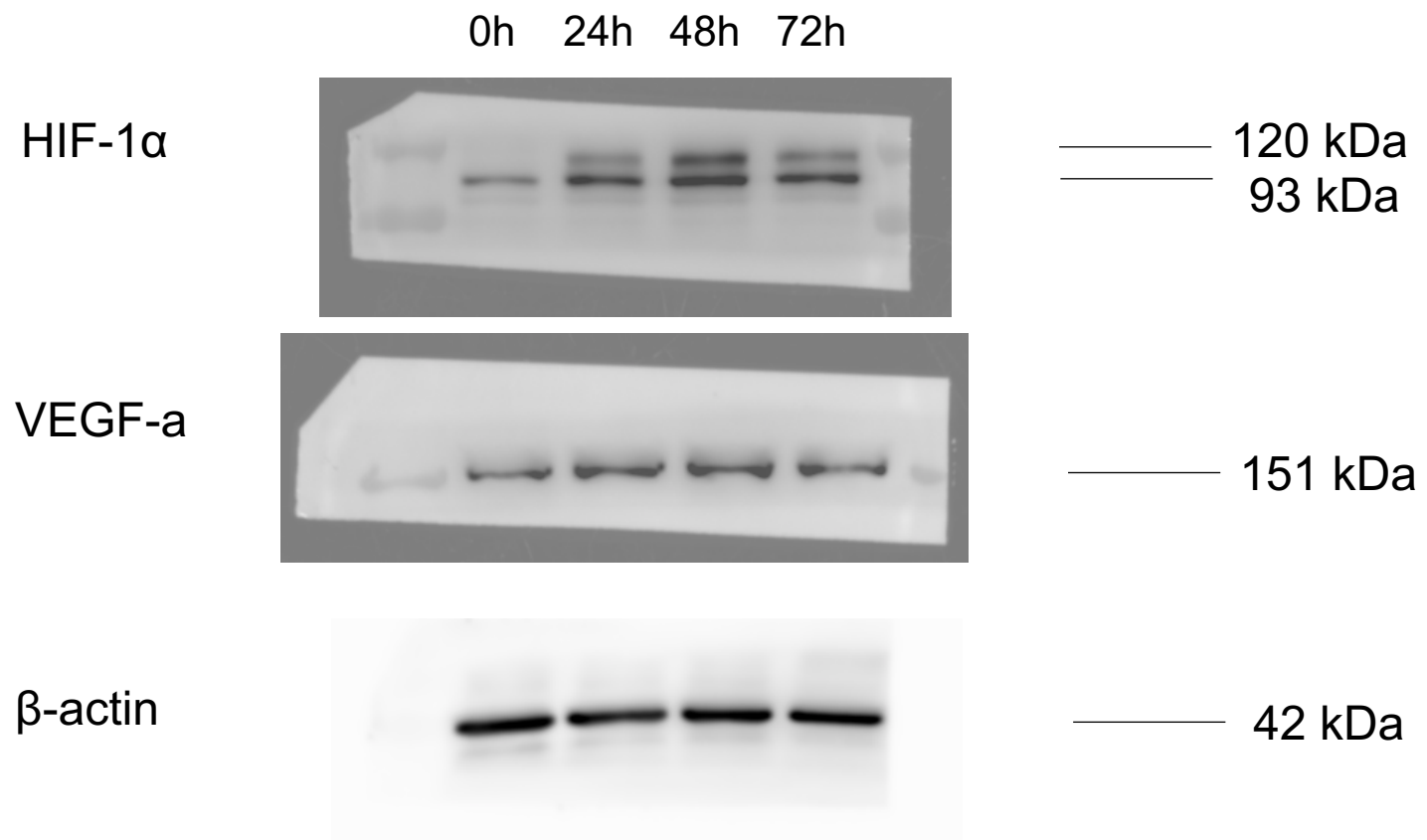

Fig 4

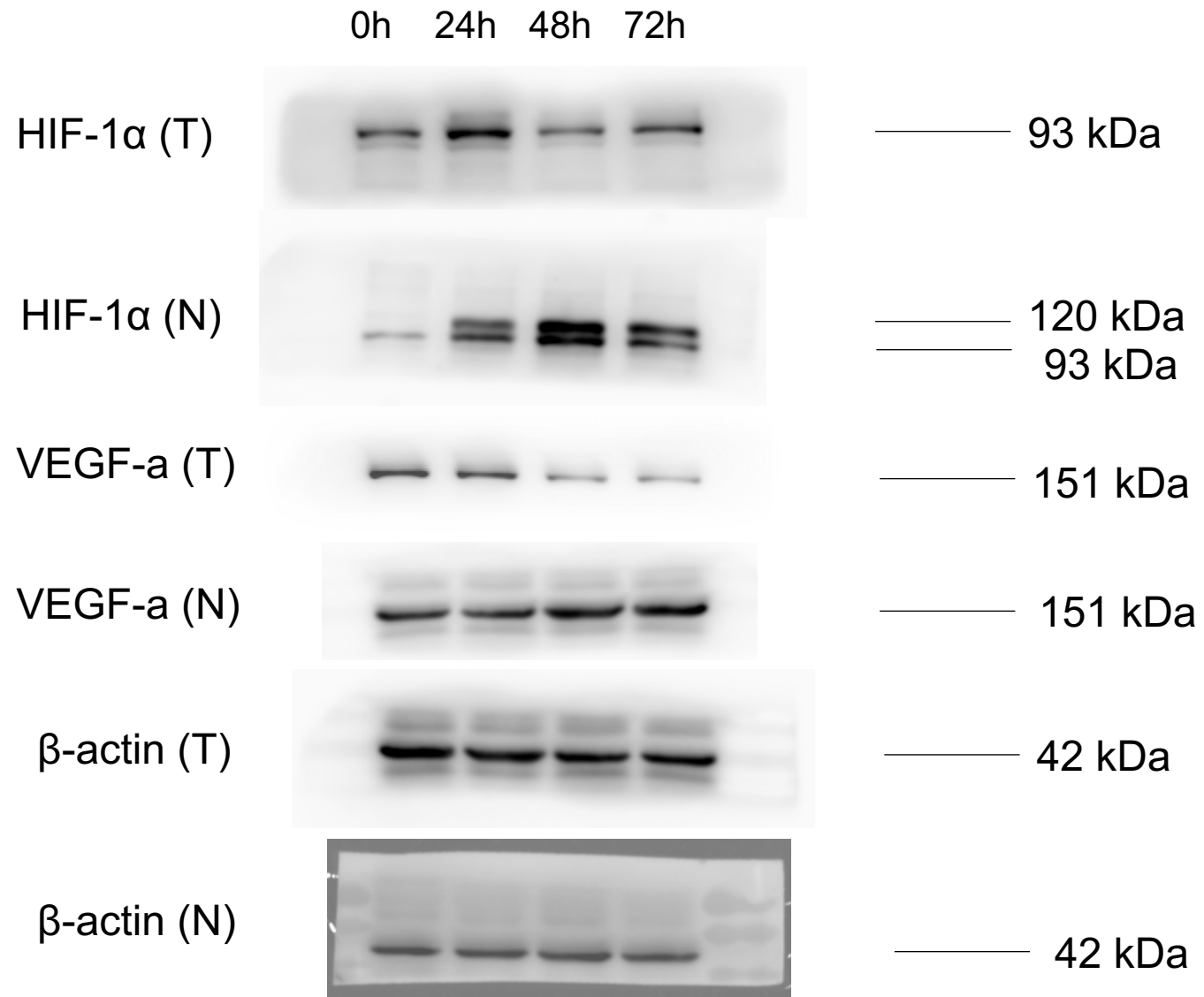

Fig 5

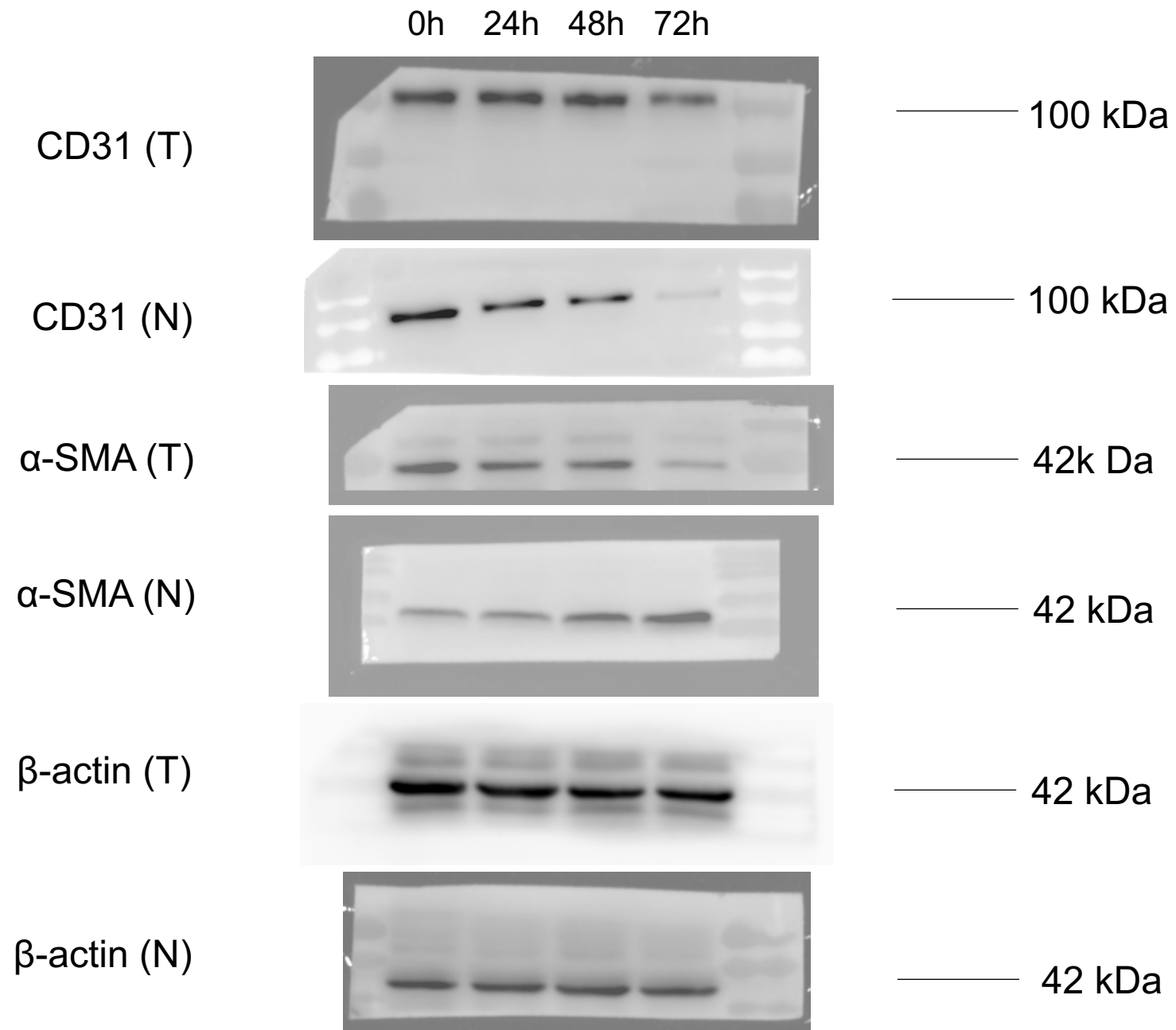

Fig 6

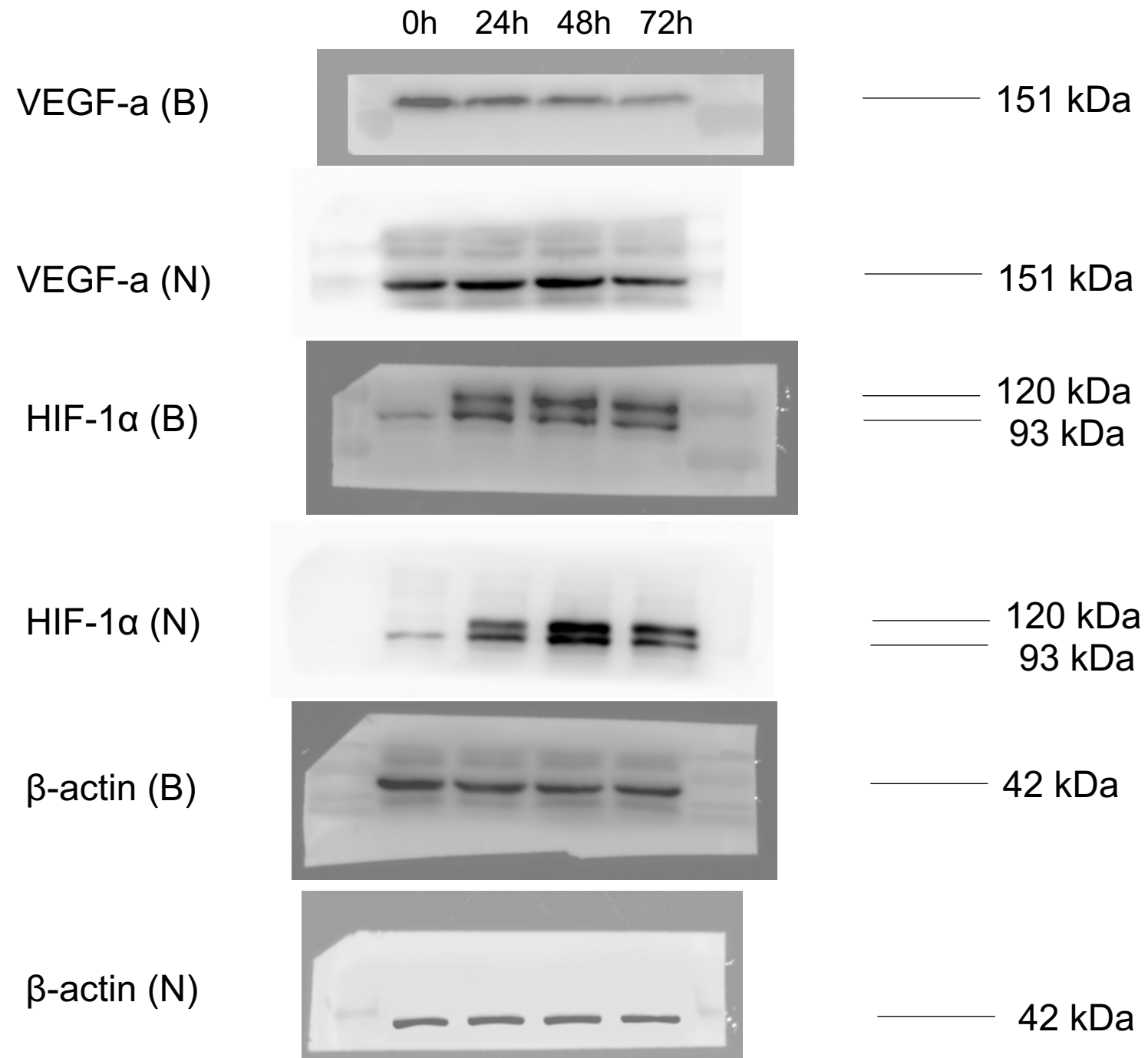

Fig 6

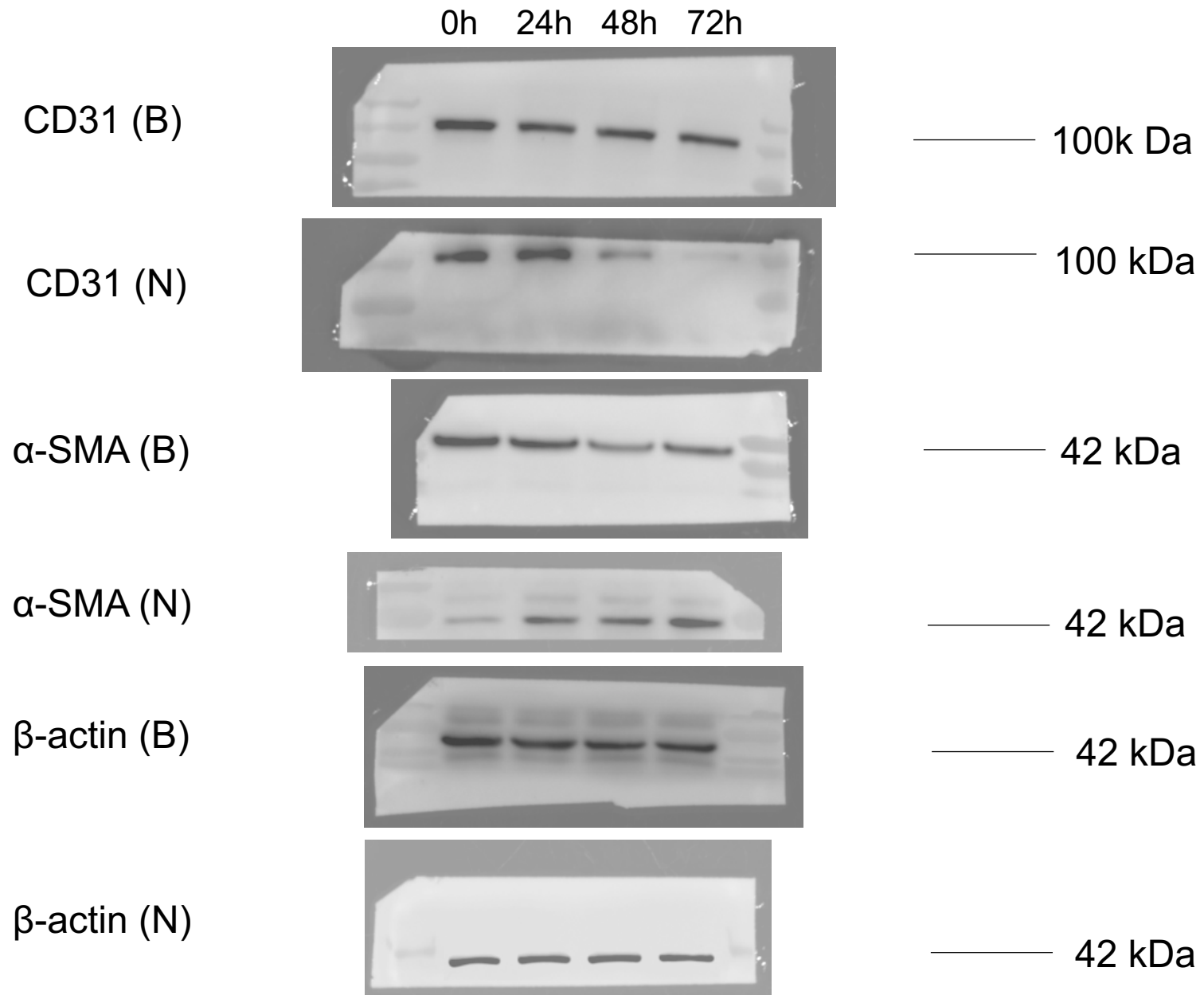

Supplement: S1 Raw images — (PDF) [file pone.0263369.s004.pdf]
